# Proteomic Profiles of Exosomes of Septic Patients Presenting to the Emergency Department Compared to Healthy Controls

**DOI:** 10.3390/jcm9092930

**Published:** 2020-09-11

**Authors:** Daniel C. Morris, Anja K. Jaehne, Michael Chopp, Zhanggang Zhang, Laila Poisson, Yalei Chen, Indrani Datta, Emanuel P. Rivers

**Affiliations:** 1 Department of Emergency Medicine, Henry Ford Hospital, Detroit, MI 48202, USA; DMorris4@hfhs.org (D.C.M.); ERivers1@hfhs.org (E.P.R.); 2 Department of Neurology Research, Henry Ford Hospital, Detroit, MI 48202, USA; MChopp1@hfhs.org (M.C.); ZZhang1@hfhs.org (Z.Z.); 3 Department of Public Health Sciences, Henry Ford Hospital, Detroit, MI 48202, USA; LPoisso1@hfhs.org (L.P.); YChen4@hfhs.org (Y.C.); IDatta1@hfhs.org (I.D.); 4 Department of Surgical Critical Care, Henry Ford Hospital, Detroit, MI 48202, USA

**Keywords:** sepsis, septic shock, exosomes, proteomic profiles, healthy volunteers, emergency department

## Abstract

Background: Septic Emergency Department (ED) patients provide a unique opportunity to investigate early sepsis. Recent work focuses on exosomes, nanoparticle-sized lipid vesicles (30–130 nm) that are released into the bloodstream to transfer its contents (RNA, miRNA, DNA, protein) to other cells. Little is known about how early changes related to exosomes may contribute to the dysregulated inflammatory septic response that leads to multi-organ dysfunction. We aimed to evaluate proteomic profiles of plasma derived exosomes obtained from septic ED patients and healthy controls. Methods: This is a prospective observational pilot study evaluating a plasma proteomic exosome profile at an urban tertiary care hospital ED using a single venipuncture blood draw, collecting 40 cc Ethylenediaminetetraacetic acid (EDTA) blood. Measurements: We recruited seven patients in the ED within 6 h of their presentation and five healthy controls. Plasma exosomes were isolated using the Invitrogen Total Exosome Isolation Kit. Exosome proteomic profiles were analyzed using fusion mass spectroscopy and Proteome Discoverer. Principal component analysis (PCA) and differential expression analysis (DEA) for sepsis versus control was performed. Results: PCA of 261 proteins demonstrated septic patients and healthy controls were distributed in two groups. DEA revealed that 62 (23.8%) proteins differed between the exosomes of septic patients and healthy controls, *p*-value < 0.05. Adjustments using the False Discovery Rate (FDR) showed 23 proteins remained significantly different (FDR < 0.05) between sepsis and controls. Septic patients and controls were classified into two distinct groups by hierarchical clustering using the 62 nominally DE proteins. After adjustment multiple comparisons, three acute phase proteins remained significantly different between patients and controls: Serum amyloid A-1, C-reactive protein and Serum Amyloid A-2. Inflammatory response proteins immunoglobulin heavy constant Δ and Fc-fragment of IgG binding protein were increased. Conclusion: Exosome proteomic profiles of septic ED patients differ from their healthy counterparts with regard to acute phase response and inflammation.

## 1. Introduction

Sepsis is the dysregulated immune host’s response to systemic infection often resulting in multi-organ dysfunction and death [1,2,3]. Each year more than 1.5 million patients present with sepsis in the United States [4]. Timely administration of intravenous fluids, antibiotics and vasoactive agents in the case of hypotension, has been the traditional treatment of sepsis; however, morbidity and mortality remain high. Exosomes are secreted small extracellular vesicles (30–100 nm) that contain protein, lipids, DNA and RNA (including mRNA, microRNA and long non-coding RNA) all of which are transferred from cell to cell to facilitate intercellular communication in both physiological and disease states [5,6]. Emerging data suggest that exosomes are involved in sepsis development and progression. A recent publication showed that the degree of organ failure and mortality in septic patients is related to higher concentrations of exosome protein content as measured via an Enzyme-linked immunosorbent assay (ELISA) [7]. Additionally, previous research showed that exosomes isolated from septic patients induce superoxide production, endothelial cell apoptosis and myocardial depression in isolated heart and papillary muscles preparations [8,9,10,11]. In addition, exosomes isolated from septic patients showed differential expression of microRNAs that were associated with the inflammatory response [12]. Other research suggests that exosomes may mitigate the inflammatory response [13]. Most research in this field has to-date been conducted in patients with established and treated sepsis and evaluated changes in targeted proteomic profiles over time. [14] Collectively, these studies suggest that exosomes from septic patients may influence the course and severity of sepsis and investigation of these exosomes may provide clues to the treatment of the disease.

The Emergency Department (ED) has become a vital link in the treatment of sepsis as early and timely administration of fluids, antibiotics and vasopressors has been shown to reduce morbidity and mortality [15]. Most studies involving exosomes in sepsis are recruited from the Intensive Care Unit (ICU); however, the clinical relevance of this pilot study is to investigate human septic exosomes recruited from the ED, the earliest possible presentation. To our knowledge a global proteomic analysis of exosomes obtained from septic patients during the earliest presentation has not been reported to date. We performed proteomic analysis of these septic exosomes and compared them with the proteomic profiles of exosomes derived from healthy volunteers. We hypothesized the exosomal proteomic profiles would differ between septic patients recruited in this earliest phase of sepsis presentation when compared to healthy controls.

## 2. Experimental Section

### 2.1. Patient Recruitment Criteria

This single-center observational pilot study at a tertiary care academic hospital was approved by the Henry Ford Hospital Institutional Review Board (IRB 11000). Consent was obtained from patients or their representative for blood draw (40 cc). We recruited 7 septic ED patients and 5 healthy controls on two separate occasions. Inclusion criteria were patients with suspected infection with the order of broad spectrum antibiotics and order of blood cultures and 2 Systemic Inflammatory Response Syndrome (SIRS) criteria (heart rate > 90 bpm, respiratory rate > 20 breath per minute or pCO_2_ > 32 mmHg, temperature < 36 °C or >38 °C and/or White Blood Cells (WBC) < 4 K/mL or >12 K/mL or >10% immature cells). At least one organ dysfunction such as lactate >2 mg/dL or hypotension with Systolic Blood Pressure (SBP) < 90 mmHg, mean arterial pressure ≤ 65 mmHg or vasopressor use and any other new end-organ dysfunction had to be present for enrollment. Exclusion criteria were age < 18 years, known pregnancy and the inability to obtain written consent from a patient or the surrogate. Patients had to be enrolled within 6 h of ED arrival.

### 2.2. Isolation and Characterization of Plasma Exosomes

Blood was collected via a singular venipuncture into standard 4 mL EDTA tubes, kept at room temperature and sample processing (centrifugation for 15 min at 2000× *g*) within 30 min following by freezing of 1 mL aliquots at −80 °C. Exosomes were isolated using the Invitrogen™ Total Exosome Isolation Kit following manufacturer’s instructions. Identification of exosomes was verified by transmission electron microscopy (TEM) as well as Western Blot detection of common exosomal proteins with antibodies (Abcam, Cambridge, MA, USA) against CD-63 (1:1000) and CD-81 (1:1000). We quantitated the exosomes by measuring the total protein concentration, assessed by the micro bicinchoninic acid (BCA) protocol (Pierce, Rockford, IL, USA).

### 2.3. Proteomics

50 µg of each exosome sample was buffered with 40 mM Tris pH 8, then reduced with 5 mM dithiothreitol and alkylated with 15 mM iodoacetamide. Excess iodoacetamide was quenched with an additional 5 mM dithiothreitol. Overnight digestion was performed with sequencing-grade trypsin (Promega, Madison, WI, USA) in 0.3 M urea and 0.3% deoxycholate. Digests were acidified with 0.5% trifluoroacetic acid and fractionated using the High pH Reversed-Phase Peptide Fractionation Kit (ThermoFisher Scientific, San Jose, CA, USA) following the manufacturers protocol. Elution buffers for fractionation consisted of 5%, 7.5%, 10%, 12.5%, 15%, 17.5%, 20% and 50% acetonitrile in 0.1% triethylamine.

High pH fractions were acidified and analyzed by reversed-phase chromatography (Acclaim PepMap100 C18 column, ThermoScientific, Waltham, MA, USA), followed by ionization with the Nanospray Flex Ion Source (ThermoScientific, Waltham, MA, USA), and introduced into a Fusion mass spectrometer (ThermoScientific Waltham, MA, USA) over 2 h. MS1 scans were collected at 120 K resolution and MS2 scans for abundant species were collected in the ion trap for 5 s following each MS1 scan.

Raw data were analyzed using Sequest (Thermo Fisher Scientific; in Proteome Discoverer 2.1.1.21, Thermo Fisher Scientific) and X! Tandem (TheGPM, thegpm.org; version CYCLONE (2010.12.01.1), Rockville, MD, USA). Sequest was set up to search the Uniprot complete human database (downloaded 14 July 2017, 20,145 entries) assuming the digestion enzyme trypsin. X! Tandem was set up to search a subset of the same database containing only proteins identified by Sequest. Sequest and X!Tandem were searched with a fragment ion mass tolerance of 0.6 Da and a parent ion tolerance of 10 PPM. Carbamidomethylation of cysteine was specified in Sequest and X!Tandem as a fixed modification. Deamidation of asparagine and glutamine, oxidation of methionine, and acetylation of the n-terminus were specified in Sequest as variable modifications. Ammonia-loss of the n-terminus, deamidation of asparagine and glutamine, oxidation of methionine, and acetylation of the n-terminus were specified in X!Tandem as variable modifications.

Scaffold (version Scaffold_4.11.0, Proteome Software Inc., Portland, OR, USA) was used to validate MS/MS based peptide and protein identifications. Peptide identifications were accepted if they could be established at greater than 98.0% probability to achieve an false discovery rate (FDR) less than 1.0% using a reversed database search. Peptide Probabilities from Sequest were assigned by the Scaffold Local FDR algorithm. Peptide Probabilities from X! Tandem were assigned by the Peptide Prophet algorithm [15,16] with Scaffold delta-mass correction. Protein identifications were accepted if they could be established at greater than 99.0% probability to achieve an FDR less than 1.0% and contained at least 2 identified peptides. Protein probabilities were assigned by the Protein Prophet algorithm [17]. Proteins that contained similar peptides and could not be differentiated based on MS/MS analysis alone were grouped to satisfy the principles of parsimony.

### 2.4. Statistical Methods

Spectral counts were used for protein quantification. Counts were normalized by total protein count per sample and log^2^ transformed. Correction of the batch standardizes the expression data through linear regression and estimates batch effects through the Empirical Bayes methods subsequently removing batch effects from the data. This was done using the ComBat method (“sva” package version 3.20, Bioconductor) [18]. This was necessary as patients had been enrolled and the proteomic analysis had occurred for two different sets (batches) of patients. Batch 1 contained 3 patients and 3 controls. Batch 2 contained 4 patients and 2 controls. Principal component analysis (PCA), along with differential expression analysis (DEA, “*limma*” package Version 3.37.10, R/Bioconductor) for sepsis versus control was completed [19]. The Benjamini–Hochberg FDR procedure was used to adjust for multiple comparisons [20]. Unsupervised hierarchical clustering was used to classify subjects based on protein levels and heatmap was used to visualize protein expressions. All the analyses were done using statistical computing program R version 3.5.0 (Bioconductor).

### 2.5. Pathway Analysis

Qiagen’s Ingenuity Pathway Analysis (IPA) was used for uploading the statistically significant proteins for core analysis which includes enrichment of canonical pathways, biological and disease network analysis (QIAGEN, Redwood City, CA, USA). This core-analysis is a feature that helps in understanding connections between significant molecules, in our case, proteins with various disease or biological processes by enrichment testing based on fisher exact test. The knowledgebase found in IPA, which makes analysis possible, is based on collection of observations made from biomedical literature. Data were analyzed through the use of IPA (QIAGEN Inc., Supplement S3) [21,22].

## 3. Results

### 3.1. Patients and Controls

Patients were identified and enrolled within less than 6 h following their initial ED presentation. Gender, age, race, hospitalization and sepsis parameters of the evaluated patients and controls are shown in Table 1. Patient’s age ranged between 41 and 81 years (average 63 years). The majority of enrolled patients were African American. For 6/7 patient’s bacterial infections were suspected and culture positivity was confirmed in 4/6 patients. One patient had suspected viral infection which was confirmed as Influenza A. One patient did not have any positive cultures but was treated for suspected septic shock pneumonia. Four patients were treated for septic shock with vasopressors at the time of enrollment. APACHEII (acute physiology and chronic health evaluation) scores [23] ranged from 11 to 34 (average 24.9) and SOFA (sequential organ failure assessment) score [24] ranged from 1 to 13 (average 7.6). This demographic and illness severity description aligns with various sepsis publications [25,26,27].

We attempted to age and gender match the control patients, with a slightly younger control group. At the time of sample collection all control subjects had normal vital signs without of signs and symptoms of potential infectious processes. More detailed clinical patient characteristics are provided in Supplemental Table S1.

### 3.2. Characterization of Exosomes

**Figure 1:**
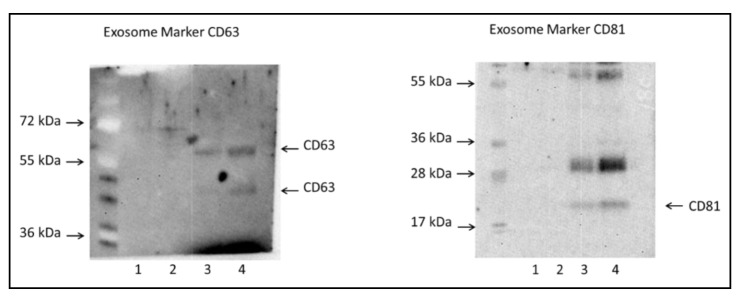
Western Blot, only septic patient samples shown.

Qualitative Western blot analysis showed the presence of CD63 and CD81 proteins in extracellular vesicles (Figure 1). CD-63 exists with varying molecular weights ranging from 30 to 60 kDa due to glycosylation) while the molecule weight of CD-81 is 25 kDa. The four lanes of each gel represent different isolation fractions with lane 4 representing the fraction that was used for proteomic analysis.

**Figure 2:**
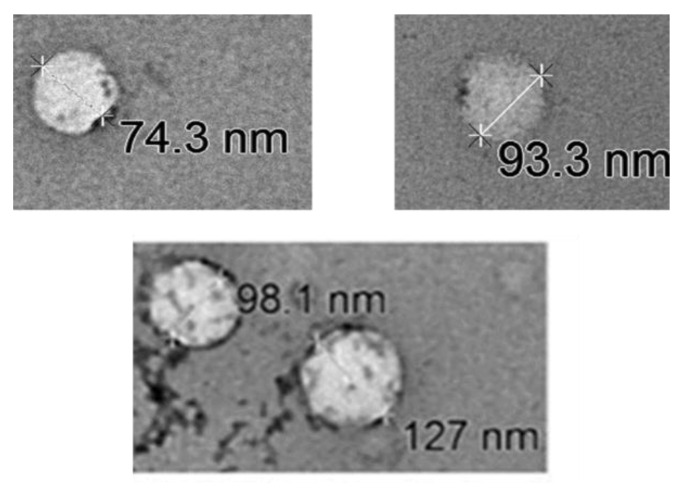
Representative Transmission Electron Microscopy images. Exosome sizes ranging from 74 to 127 nm at a maximal magnification of 90.000 via direct magnification.

Transmission electron microscopy (TEM) showed that sizes of extracellular vesicles ranged from 74 to 127 nm (Figure 2) suggesting that these extracellular vesicles are exosomes (Figure 2). No difference was seen when comparing exosomes of patients with sepsis and healthy controls.

### 3.3. Proteomics: Comparison of Control vs. Sepsis Patients: Batch Effect Correction

**Figure 3:**
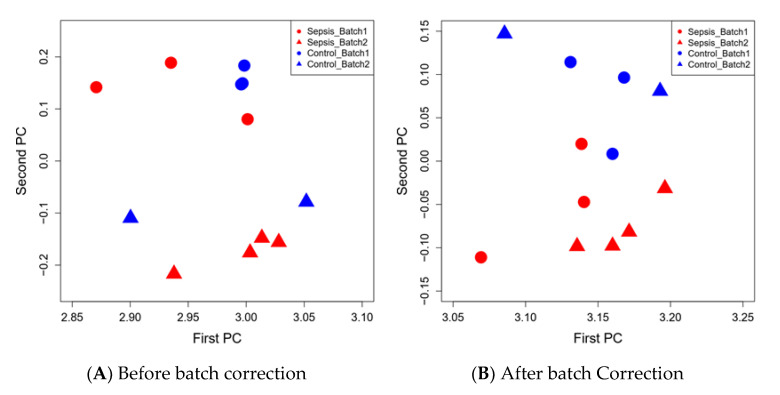
Plot of first two batches from Principal Component Analysis (PCA) of 261 proteins before (**A**) and after (**B**) batch correction. Blue color indicates controls and red color indicates sepsis. Circle represents first batch and triangle represents second batch. Before batch correction (**A**), patients in first batch (circle) are clearly separated from patients in second batch (triangle).

For this pilot project patients and controls were recruited on two separate occasions. In total, 261 total proteins were identified. Batch effect was obvious based on the Principal Component Analysis (PCA) (Figure 3A), *ComBat* method was used to estimate and remove batch effect. PCA using the 261 proteins after batch correction was then conducted and the top two PCs accounted for 93.3% of variance in the data. The plot of the first and second PCs showed that batch effect was greatly reduced, and most control and sepsis patients were clustered into two distinct groups (Figure 3B). Raw data are provided for two batches in Supplemental Tables S4 and S5.

### 3.4. Differential Expression Analysis of Sepsis vs. Controls

**Figure 4:**
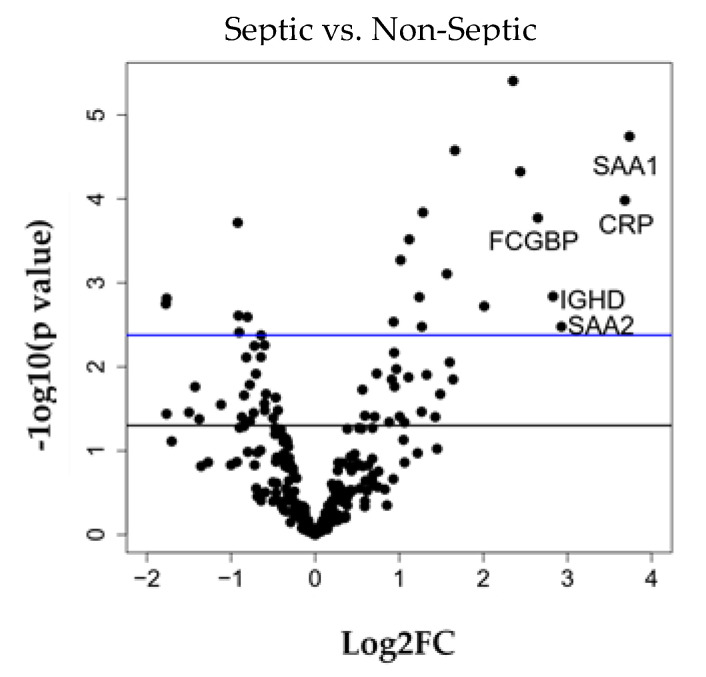
Volcano plot of 26fig1 batch corrected proteins. X-axis: log2 fold change of sepsis versus control; y-axis: −log10 (*p* value). Black horizontal line indicates *p* value = 0.05 and the blue horizontal line indicates FDR = 0.05. Among the 23 significant proteins after false discovery rate (FDR) correction the top five proteins with largest fold changes are labeled in the plot.

In the differential expression analysis (DEA) comparing sepsis vs. controls, 62/261 proteins reached significance with a nominal *p* value < 0.05 (28 expressed higher in control and 34 expressed higher in sepsis, Figure 3). Of these 62 proteins, 23 proteins remained significant after using the FDR-method for adjusting multi-comparisons at a significant level of FDR < 0.05 (Figure 4). The following 5 of 23 proteins that remained significant after multi-comparison adjustment showed that the biggest fold changes between septic patients and controls: Serum amyloid A-1 (SAA-1) (3.74 fold-increase), C-reactive protein (CRP) (3.68 fold-increase), Serum Amyloid A-2 (SAA-2) (2.93 fold-increase), Immunoglobulin heavy constant Δ (IGHD) (2.83 fold increase) and Fc-fragment of IgG binding protein (FCGBP) (2.65 fold-increase).

**Figure 5:**
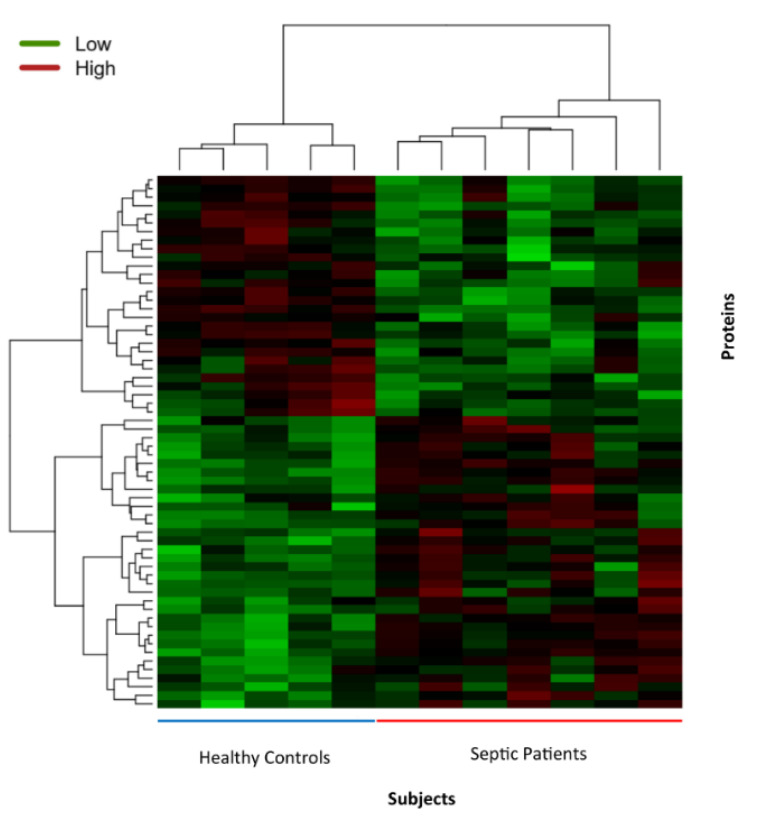
Heat map of 62 differently expressed proteins between patients with sepsis and healthy controls. Each column represents one subject and each row represents one protein. Top dendrogram shows hierarchical clustering of study subjects and left dendrogram shows hierarchical clustering of proteins. The red color indicates higher expression of proteins and the green color indicates lower expression of proteins and color scale was centered for each row/protein to allow better visualization of expression difference of a protein across samples.

Using the 62 nominally differentiated proteins in sepsis and controls, unsupervised hierarchical clustering classified the subjects into two groups where one group contained only septic patients and the other contained only controls. Heat map representation of these results is shown in Figure 5.

### 3.5. Enrichment Analysis of Proteomic Data

Acute phase signaling was top canonical pathway enriched (enrichment *p* value = 0.01) with these significant proteins, and this pathway is related to an inflammatory response which can be triggered through infection as well as other factors such as tissue injury, trauma or surgery, neoplastic growth or immunological disorders, which lead to an increase in inflammatory factors such as pro-inflammatory cytokines. Some of the key proteins in this canonical pathway were α-2-macroglobulin (A2MG), fetuin-1 protein (FETUA), albumin (ALBU), apoprotein A1 (APOA1), CO_2_, C-Reactive Protein (CRP), Factor VIII (FA8), histidine phosphotransferase (HPT), inter-α-trypsin inhibitor heavy chain J2 (ITIH2), inter-α-trypsin inhibitor heavy chain H3 (ITIH3), plasma kallikrein (KLKB1), lipopolysaccharide binding protein (LBP), serum amyloid A1 (SAA1), serum amyloid A2 (SAA2), HEP2 protein (HEP20, outer arm dynein intermediate chain 1 (IC1), serum iron transport protein transferrin (TRFE), transthyretin (TTHY) and von Willebrand factor (vWF). In addition, this pathway is predicted to be activated with a positive z score of 2.6. An enrichment of inflammatory response as a biological function network was also identified, this network is comprised of 3 sub biological networks (Degranulation of cells, *p* value = 0.001, Degranulation of blood platelets *p* value = 0.011, Degranulation of phagocytes *p* value = 0.0379) (genes involved in these networks are shown in Supplement S3. Proteomics biological network).

## 4. Discussion

Our pilot study is the first to describe a global proteomic evaluation of exosomes isolated from septic patients at the time of early presentation to the ED (<6 h). The variable timeframe of symptoms prior to ED presentation is common in patients with community acquired sepsis and the enrollment within less than 6 h from ED presentation represents a uniquely early examination of proteomic exosome profiles in contrast to studies enrolling patients in the intensive care unit within 24 h following admission to the hospital [28]. To our knowledge this is the first study looking at exosomal protein profiles at this early clinically relevant time point. Our previous work describing early variations of free circulating cytokines has shown dramatic changes in the early phases of sepsis [29,30]. It is generally accepted that rapid diagnosis and treatment of sepsis leads to improved outcomes, with regard to morbidity and mortality, and may reduce health resource consumption [15]. Early treatment implementation may contribute to cytokine changes and, therefore, sample collection at a very early time point is crucial [29,31]. Isolation and evaluation of exosomes in this early phase before hospital and intensive care admission provides information on the possible underlying mechanisms of multiple organ dysfunction.

The results indicate three specific proteins whose levels are significantly elevated above all others, SAA-1, CRP, SAA-2 found in exosomes of septic patients. These proteins are involved in the acute phase response (APR). The APR coincides with infection or tissue injury and is usually a systemic reaction involving fever, leukocytosis and increased serum levels of acute phase proteins (APPs), CRP and serum amyloid components. APPs are rapidly synthesized in the liver. They are potent opsonins and can activate the innate immune system, in particular neutrophils [32]. Due to either pro or anti-inflammatory functions their exact role during sepsis is still not well understood [33]. All relevant articles concerning APPs in sepsis refer to free circulating APPs and have not yet reported concern as components of exosomes in septic patients.

Free circulating CRP has been extensively studied in various settings of inflammation including autoimmune diseases and infection. During bacterial infections CRP can bind to polysaccharides such as polycholine on microorganisms which triggers the classical innate complement pathway with activation of C1q [34,35]. Transcriptomic upregulation of CRP is induced by increased levels of interleukin-6, which is commonly elevated in the early phases of sepsis [30]. The structurally of CRP as a homopentameric protein relates to SAA. In this concern it is important to note that CRP has been indicated as mediator of atherosclerosis in cardiovascular diseases directly inducing genes that trigger monocyte adhesion along with intracellular recruitment of E-selectin and monocyte chemoattractant protein-1 [36]. The stimulation of atherosclerotic cells via CRP triggers apoptosis and increases apoptotic plaque size [34].

Similar to CRP, free circulating SAA-1 and SAA-2 are APR reactants primarily expressed in the liver but can also be expressed in endothelial cells, smooth muscle cells, monocytes and macrophages [37]. The function of SAA is not well understood. A variety of effects and functions have been described such as detoxification of endotoxin, immune response depression, inhibition of lymphocyte and endothelial cell proliferation, inhibition of platelet aggregation, neutrophil activities and inhibition of T-cell adhesion to extracellular matrix proteins among other functions [37]. These functions highlight the importance of these APP in the overall modulation of the immune system.

We speculate that exosomes in early sepsis contain higher amounts of APP to stimulate specific immune responses either via a humoral pathway or direct immune cell stimulation to aid the resolution of the immune dysregulation. With this exosome proteomic evaluation could be a potential assessment tool for sepsis in the early phases of sepsis.

The other two highly expressed proteins that we observed in sepsis exosomes, immunoglobulin heavy constant Δ (IGHD) and Fc-fragment of IgG binding protein (FCGBP) are components of the inflammatory response. The IGHD activates B-cells and once activated are able to produce antibodies in response to infection [38]. Werdan described that administration of intravenous immunoglobulins neutralizes and opsonizes antibodies and with this increase serum bactericidal activity, stimulates phagocytosis of leukocytes and neutralizes pathogens and their virulence factors [39,40]. The Fc-fragment of the antibody interacts with Fc-receptors on the cell surface as well as proteins of the complement system which activates the immune system. Levels of these two immunologic proteins are highly elevated in sepsis exosomes suggesting a role in the acute early inflammatory response. Supplemental immune globulins have been proposed as a possible treatment in critical ill septic patients. Exosomes may be able to deliver targeted IgG therapies to further modulate the immune system.

The results of our project are consistent with other studies investigating the role of exosomes in sepsis. Real et al. isolated exosomes from patients with sepsis and observed significant changes in miRNAs when compared to controls [12]. Most of these changes were related to the inflammatory response. Additionally, they observed that miRNAs packed in exosomes from sepsis patients who survived, were associated with pathways related to cell cycle regulation suggesting that reentry into the proliferative or differentiation cell cycle phase was important for recovery.

This pilot study has various limitations. First, the sample size of patients and controls was small, and was not specifically matched for age, race or co-morbities. Secondly, rapid hemodynamic but also general proteomic and inflammatory changes occur in the early phases of sepsis. To address these changes, further studies need to also investigate the temporal pattern of these proteomic changes. However, these results provide indications for further investigations and highlight the feasibility and stability of the results using plasma exosomes in two separate batches. This initial investigation gives guidance for future studies evaluating the mechanistic role exosomes play in sepsis.

## 5. Conclusions

In summary, we performed proteomic analysis of plasma exosomes from septic ED patients and compared these to the proteomic profile of controls. Our results show that APP and immunoglobulins involved in the inflammatory response are upregulated in septic exosomes. This suggests that exosomes are involved in sepsis. Further research is warranted to determine if exosomes are beneficial or detrimental in the pathogenesis of sepsis and if these may be useful for the diagnosis and as targeted treatment vehicles.

## Figures and Tables

**Table 1:** Patient Characteristics.

	Batch 1						Batch 2					
	Patient 1	Patient 2	Patient 3	Control 1	Control 2	Control 3	Patient ATB1	Patient ATB2	Patient ATB24	Patient ATB46	Control 1	Control 2
**Gender**	Male	Female	Female	Male	Female	Female	Male	Male	Male	Male	Male	Male
**Age**	61	74	52	55	24	39	64	81	68	41	60	54
**Race**	African American	African American	Caucasian	Caucasian	Asian	Caucasian	African American	African American	African American	Unknown	African American	African American
**Primary Source**	Blood	Lung, Free Intraabdominal air	Urogenital	None	None	None	Lung	Abdomen	Lung, peritoneal dialysis cath	Blood	None	None
**Sepsis Class**	Shock	Shock	Severe	Healthy	Healthy	Healthy	Severe	Severe	Shock	Shock	Healthy	Healthy
**30 Day Outcome**	Dead	Alive	Alive	Alive	Alive	Alive	Alive	Alive	Alive	Dead	Alive	Alive
**Length of Stay**	17	7	4				6	6	28	11		
**Day 1 SOFA Score**	13	9	14				4	4	12	10		
**Day1 APACHE Score**	24	33	15				11	30	34	27		
**Vasopressor Use**	Yes	Yes	No				No	Ni	Yes	Yes		
**Culture Positive**	Wound	Urine, Blood	Urine				Nasal Swab	Urine	None	None		
**Organism**	Pseudomonas aeruginosa	Candida albicans, Staph Coag (-)	Escherichia Coli				Influenza A	Enterococcus Sp.	None	None		
**Highest Lactate mg/ dL**	10.8	10.0	4.5				8.5	1.1	3.1	1.6		

## Data Availability

The mass spectrometry proteomics data have been deposited to the ProteomeXchange Consortium (http://proteomecentral.proteomexchange.org) via the MassIVE partner repository with the dataset identifier PXD020106.

## Supplementary Materials

The following are available online at https://www.mdpi.com/2077-0383/9/9/2930/s1, Supplement Table S1: Patient Characteristics; Supplement S2. IPA Knowledge Base letter; Supplement S3. Acute Phase Response Signaling; Supplement Table S4: Protein Quantification for Batch 1; Supplement Table S5: Protein Quantification for Batch 2.
