# Supplementary material for: Proteomic Profiles of Exosomes of Septic Patients Presenting to the Emergency Department Compared to Healthy Controls
Source: J Clin Med. 2020 Sep 11;9(9):2930. doi: 10.3390/jcm9092930 (PMC7564089; doi:10.3390/jcm9092930)
Supplement: Supplementary file 1 [file jcm-09-02930-s001.zip › JCM_Supplemental Data/S1_Supplement S1_Table S1. Patient Characteristics.pdf]

	Patient 1 (B1)	Patient 2 (B1)	Patient 3 (B1)	Control 1 (B1)	Control 2 (B1)	Control 3 (B1)	Patient 1 (B2)	Patient 2 (B2)	Patient 3 (B2)	Patient 4 (B2)	Control 1 (B2)	Control 2 (B2)
**Gender**	Male	Female	Female	Male	Female	Female	Male	Male	Male	Male	Male	Male
**Age**	61	74	52	55	24	39	64	81	68	41	60	54
**Race**	African American	African American	Caucasian	Caucasian	Asian	Caucasian	African American	African American	African American	Unknown	African American	African American
**Primary Source**	Blood	Lung, Intra-abdominal	Urogenital	NONE	NONE	NONE	Lung	Abdomen	Lung, Intra-abdominal	Blood	NONE	NONE
**Sepsis Class**	Shock	Shock	Severe	Healthy	Healthy	Healthy	Severe	Severe	Shock	Shock	Healthy	Healthy
**30 Day Outcome**	Died	Alive	Alive	Alive	Alive	Alive	Alive	Alive	Alive	Died	Alive	Alive
**Length of Stay**	17	7	4				6	6	28	11		
**Day 1 SOFA Score**	13	9	1				4	4	12	10		
**Day 1 APACHE S**	24	33	15				11	30	34	27		
**Culture Positive?**	Wound	Urine, Blood	Urine				Nasal Swab	Urine	None	None		
**Organism**	P. aeruginosa	C. albicans, Staph, coag (-)	E. Coli				Influenza A	Enterococcus sp.	None	None		
**Vasopressor Use**	Yes	Yes	No				No	No	Yes	Yes		
**Highest Lactate**	10.8	10	4.5				8.5	1.1	3.1	1.6		

Supplemental Table S1. Patient Characteristics
