# Supplementary material for: Proteomic Profiles of Exosomes of Septic Patients Presenting to the Emergency Department Compared to Healthy Controls
Source: J Clin Med. 2020 Sep 11;9(9):2930. doi: 10.3390/jcm9092930 (PMC7564089; doi:10.3390/jcm9092930)
Supplement: Supplementary file 1 [file jcm-09-02930-s001.zip › JCM_Supplemental Data/S2_Supplement S2_IPA Knoweldge Base letter.pdf]

**QIAGEN Bioinformatics Technical Support** April 1, 2020

To whom this may concern:

Dr. Daniel C. Morris has been granted permission by QIAGEN Silicon Valley to use copyrighted figures (both print and digital) generated from Ingenuity Pathway Analysis (IPA) for his publication.

Figures produced from IPA are available under an open-access CC-BY license for purposes of publication.

If you have any further questions, please contact QIAGEN Bioinformatics Technical Support at ts-bioinformatics@qiagen.com .

Best regards,
(on behalf of QIAGEN Silicon Valley)

Ana Katrina David
Scientist, Global Bioinformatics Technical Services

QIAGEN - Sample to Insight

Phone American: +1-866-464-3684 | Danish: +45-80 82 0167
ts-bioinformatics@qiagen.com
www.qiagenbioinformatics.com

Making improvements in life possible.
